# Insulin Signaling in Liver and Adipose Tissues in Periparturient Dairy Cows Supplemented with Dietary Nicotinic Acid

**DOI:** 10.1371/journal.pone.0147028

**Published:** 2016-01-14

**Authors:** Asako Kinoshita, Ákos Kenéz, Lena Locher, Ulrich Meyer, Sven Dänicke, Jürgen Rehage, Korinna Huber

**Affiliations:** 1 Department of Physiology, University of Veterinary Medicine Hannover, Foundation, Hannover, Germany; 2 Institute of Animal Science, University of Hohenheim, Stuttgart, Germany; 3 Clinic for Ruminants with Ambulatory and Herd Health Services at the Center of Veterinary Clinical Medicine, Ludwig-Maximilians-University Munich, Germany; 4 Institute of Animal Nutrition, Federal Research Institute for Animal Health, Friedrich-Loeffler-Institute, Braunschweig, Germany; 5 Clinic for Cattle, University of Veterinary Medicine Hannover, Foundation, Hannover, Germany; University of Catanzaro Magna Graecia, ITALY

## Abstract

The glucose homeostasis in dairy cattle is very well controlled, in line with the metabolic adaptation during the periparturient period. Former studies showed that nicotinic acid (NA) lowered plasma non-esterified fatty acids (NEFA) concentrations and increased insulin sensitivity in dairy cows. Thus, the purpose of this study was to investigate whether the expression of proteins involved in hepatic and adipose insulin signaling and protein expression of hepatic glucose transporter 2 (GLUT2) were affected by dietary NA and dietary concentrate intake in periparturient dairy cows. Twenty pluriparous German Holstein cows were fed with the same diet from about 21 days before the expected calving date (d-21) to calving. After calving, cows were randomly assigned in 4 groups and fed with diets different in concentrate proportion (“HC” with 60:40% or “LC” with 30:70% concentrate-to-roughage ratio) and supplemented with NA (24 g/day) (NA) or without (CON) until d21. Biopsy samples were taken from the liver, subcutaneous (SCAT) and retroperitoneal (RPAT) adipose tissues at d-21 and d21. Protein expression of insulin signaling molecules (insulin receptor (INSR), phosphatidylinositol-3-kinase (PI3K), protein kinase Cζ (PKCζ)) and hepatic GLUT2 was measured by Western Blotting. The ratio of protein expression at d21/at d-21 was calculated and statistically evaluated for the effects of time and diet. Cows in HC had significantly higher dietary energy intake than cows in LC. In RPAT a decrease in PI3K and PKCζ expression was found in all groups, irrespectively of diet. In the liver, the GLUT2 expression was significantly lower in cows in NA compared with cows in CON. In conclusion, insulin signaling might be decreased in RPAT over time without any effect of diet. NA was able to modulate hepatic GLUT2 expression, but its physiological role is unclear.

## Introduction

The periparturient period is critical for the occurrence of metabolic disorders associated with severe energy deficit and excessive lipolysis in dairy cattle [1]. High yielding dairy cows are at high metabolic burden, especially in early lactation due to increasing energy demands for milk production which cannot be covered by dietary intake. Consequently, cows physiologically undergo a drastic shift in metabolism from anabolic to catabolic status around parturition. In the catabolic status after calving, lipolysis and accumulation of triacylglycerides in the liver take place [1]. Insulin blood concentration and responsiveness of peripheral tissues to insulin are lowest in this period, so that shifting of glucose into the mammary gland for the milk synthesis is maximized [2]. In comparison to humans and monogastric animals, dairy cows have physiologically much lower glucose concentrations in the blood and are known to be less insulin sensitive [2]. On a molecular level, the protein and mRNA expression of insulin-dependent glucose transporter 4 (GLUT4) were lower in muscles of ruminating animals compared to non-ruminating animals [3,4]. The lower expression of this key component of insulin-dependent glucose uptake may contribute to the constitutively lesser insulin sensitivity of ruminants.

The high non-esterified fatty acids (NEFA) blood concentrations in cows with metabolic disorders is an indicator for an excessive lipolysis. It is likely to be based on the reduced insulin sensitivity and thereby decreased anti-lipolytic effect of insulin at least in part [1]. It is suggested that dietary nicotinic acid (NA) could act as an alternative anti-lipolytic agent. NA suppresses lipolysis by reducing cyclic AMP (cAMP) similarly to insulin, but by a different pathway via inhibition of adenylate cyclase through activation of NA receptor (GRP109A) also in dairy cows [5,6]. In vivo application of NA by i.v. infusion reduced plasma concentrations of NEFA, which led to an increased response to endogenous insulin measured by glucose and insulin tolerance tests [7]. These effects of NA on insulin sensitivity can be affected by dietary energy supply and rate of lipolysis in cows [7]. Moreover, absorption and metabolism of dietary supplemented NA and its effects on performance could differ dependently on the composition of the diets [8].

Therefore, a trial was conducted to assess—besides anti-lipolytic action—potential effects of NA on hepatic and adipose insulin signaling and glucose transporter expression. Primiparous and pluriparous cows were fed diets with two different concentrate proportions with or without NA supplementation over the entire lactation period [9]. Of these, a subset of cows that were randomly selected and were used for the present study. Using samples from liver and two different adipose depots, we investigated protein expression of key components of insulin signaling. Moreover, in the liver we investigated the expression of glucose transporter 2 (GLUT2). The function of the SLC2A2 gene product, GLUT2, is to facilitate glucose release from mammalian hepatocytes [10]. The expression of GLUT2 was also discussed in dairy cows as an indicator for hepatic glucose secretion [11,12]. This study aimed to test the hypothesis that the expression of the selected proteins was affected by the onset of lactation, dietary NA supplementation, and dietary concentrate proportion. So far, molecular mechanism of regulation of insulin signaling in dairy cows has not been extensively investigated at protein level.

## Materials and Methods

### Animals and feeding

The experiment was conducted according to the European Community regulations concerning the protection of experimental animals and the guidelines of the Lower Saxony State Office for Consumer Protection and Food Safety (LAVES), Oldenburg, Germany (File number 33.14-42502-04-085/09). All the necessary assessments regarding the protection of animal welfare and ethics during the animal experiment was conducted by LAVES. The feeding trials of cows were performed in 2010 at the experimental station of the Institute of Animal Nutrition, Friedrich-Loeffler-Institute (FLI), Braunschweig, Germany. Cows used in this study were before, during and after the experiments a part of a dairy cow herd of FLI. Briefly, 20 pluriparous German Holstein cows were dried off 8 weeks before calving and received a silage-based diet (40% grass silage and 60% corn silage on a dry matter (DM) basis). From about three weeks before to the expected calving date (d-21) cows were fed a diet containing concentrate at 15% of DM of total ration until partum. On the day after calving, cows were assigned randomly to four groups (n = 5/group). Two groups of them were fed diets with NA supplementation (NA) (> 99.5% nicotinic acid, not rumen-protected, Lonza Ltd., Basel, Switzerland, 24 g/day per animal) and the other two groups were fed a control diet (CON) until 21 days after calving (d21). Within NA and CON each, one group received a diet with a high concentrate proportion (“HC” with 60:40% concentrate-to-roughage ratio), and another group received a diet with a low concentrate proportion (“LC” with 30:70% concentrate-to-roughage ratio). Diets for NA-LC and CON-LC comprised 42% corn silage and 28% grass silage, whereas the diet for NA-HC and CON-HC comprised 24% corn silage and 16% grass silage. The animals were kept in a freestall housing system with free access to water. The roughage (maize and grass silage) and concentrate were fed *ad libitum* separately through an automatic feeding station (“Type MJ/RIC” for roughage feeding and “Type MJ” for concentrate feeding, Insentec, B. V., Marknesse, the Netherlands). The available amount of the concentrate was adjusted twice a week individually in order to realize the intended concentrate-to-roughage ratio. Daily feed intake was recorded automatically via ear transponders worn by all the cows (Insentec B. V.). Representative concentrate samples and maize and grass silage samples were analyzed according to the protocols of the Association of German Agricultural Analysis and Research Centres [13]. Milk yield and body weight after milking were recorded automatically (Lemmer Fullwood GmbH, Lohmer, Germany) daily. The actual intake of NA was 1.0 ± 0.0, 2.8 ± 0.03, 20.5 ± 0.2, and 21.0 ± 0.3 g/d for CON-LC, CON-HC, NA-LC, and NA-HC, respectively [9]. Nutrient, energy and fiber contents of the silages and concentrate as well as the composition of the diet fed to each experimental group post partum are presented as supporting information (S1 Table).

### Blood Sampling and Analysis

Blood samples were taken at 7:30 after a feed withdrawal of at least 2 h from the jugular vein at d-21 and d21. Samples were centrifuged at 2000 *g* for 10 min and serum and plasma were stored at -80°C until analysis. Plasma glucose and serum NEFA concentration were determined using an automatic analyzer system (Cobas Mira, F. Hoffmann-La Roche Ltd., Basel, Switzerland). The serum insulin concentration was determined by RIA (IM3210, Immunotech, Beckman Coulter Inc., Bera, USA). Revised quantitative insulin sensitivity check index (RQUICKI, RQUICKI = 1/[log(Gluc) + log(Ins) + log(NEFA)]) was calculated according to Holtenius and Holtenius [14]. Plasma concentration of nicotinamide was determined via HPLC as described by Niehoff et al. [15].

### Sampling of Hepatic and Adipose tissue

Biopsy sampling from the liver, the subcutaneous adipose tissue (SCAT) and the retroperitoneal adipose tissue (RPAT) was performed at d-21 and d21. The cows were fixed at the station used for claw treatments. The area of each biopsy sampling point was shaved, washed, defatted and disinfected with 70% ethanol and iodine. Infiltration anesthesia with 5 mL procaine (Selectavet, Weyarn-Holzolling, Germany) was applied. For sampling of hepatic tissue biopsies a skin incision of 1 cm length was made at the 9^th^ or 10^th^ intercostal space with a scalpel. The incision was then punctured with a cannula with 3 mm width and 2 cm length. The liver biopsy was taken through this skin hole with an automatic device for biopsy sampling and commercial Tru-Cut biopsy needle (Bard Magnum, Tru-Cut 12G needle, Bard Biopsy Systems, USA). The location of biopsy sampling was confirmed by tracking the needle via ultrasonography. For the sampling of SCAT, a 1.5 cm skin incision was made in the region of the tail head. To obtain the RPAT, a 3 cm skin incision was made in the angle between the lumbar transversal processus (about 5 cm ventrally) and iliac bone (about 5 cm cranially), muscles were dissected reaching the peritoneum, and adipose tissue biopsies were taken directly above the peritoneum [16]. After the sampling, the wounds were closed by a suture (Filovet, Wirtschaftsgenossenschaft Deutscher Tierärzte, Garbsen, Germany), treated with aluminum-spray and controlled every day until healing.

### Sample Preparation

About 25 mg of hepatic tissues and 50 mg of adipose tissues were ground in liquid nitrogen, homogenized in 1 mL lysis buffer [in m*M*: 50 HEPES, pH 7.4; 0.1% Triton X-100 (vol/vol); 4 ethylene glycol-bis(2-amino-ethylether)-N,N,N´,N´-tetraacetic acid (EGTA); 10 EDTA; 100 β-glycerophosphate; 15 tetrasodium pyrophosphate; 5 sodium orthovanadate; 2.5 sodium fluoride] containing protease inhibitors (cOmplete, Mini, F.Hoffmann-La Roche Ltd., Basel, Switzerland) and incubated for 1 h at 4°C under continuous shaking. After incubation, the mixture was further homogenized with a 22-gauge needle and frozen in 100 μL aliquots at -20°C until electrophoresis. Protein concentrations of the homogenates were determined using protein assay according to Bradford (Bradford Reagent, 5 ×, SERVA, Heidelberg, Germany).

### Electrophoresis, Western Blot

Forty (liver) or 20 (adipose tissues) micrograms of protein were diluted in Laemmli buffer (50 m*M* Tris-HCl, 10% glycerol (vol/vol), 5% SDS (wt/vol), bromophenol) [17] and mercaptoethanol (2% (vol/vol)) in a total volume of 20 μL and were heat-denatured at 95°C for 5 min and then applied to SDS-PAGE. Electrophoresis was run at 60 V for 30 min in stacking gels (5% acrylamide (vol/vol), 125 m*M* Tris-HCl, pH 6.8, 0.1% SDS (wt/vol), 0.1% APS (wt/vol), 0.1% TEMED (vol/vol)) followed by 120 V for 90 min in resolving gels (8% acrylamide, 375 m*M* Tris-HCl with pH 8.8, 0.1% SDS, 0.1% APS, 0.06% TEMED) using running buffer (250 m*M* Tris-HCl, 190 m*M* glycine, and 10% SDS).

Protein was transferred to nitrocellulose membrane (Bio-Rad Laboratories, Inc., Hercules, USA) by means of electrophoretic blotting using a tank system (Mini-Protein Tetra Cell, Bio-Rad Laboratories, Inc.). Transfer was performed at 100 V for 90 min in transfer buffer (250 m*M* Tris-HCl, 190 m*M* glycine, and 20% methanol (vol/vol)). Membranes were treated with blocking buffer containing 5% skimmed milk-phosphate-buffered saline (PBS) (137 m*M* sodium chloride, 2.7 m*M* potassium chloride, 1.8 m*M* monopotassium phosphate, and 10.1 m*M* disodium phosphate) + 0.1% Tween 20 (vol/vol) at room temperature for 60 min, and afterwards three washing cycles with PBST (PBS + 0.1% Tween 20) of 5 min each. Membranes were incubated with antibodies raised in rabbits against glucose transporter 2 (GLUT2) (GT21-A, Alpha Diagnostic Intl. Inc., San Antonio, USA, diluted at 1:100 in 2.5% skimmed milk-PBST), phosphatidylinositol-3-kinase (PI3K) p85α subunit (sc-423, SantaCruz Biotechnology, Inc., Dallas, USA, diluted at 1:200 in 2.5% BSA-PBST) and insulin receptor (INSR) β subunit (sc-711, SantaCruz Biotechnology, Inc., diluted at 1:400 in 2.5% skimmed milk-PBST) for liver and with antibodies raised in rabbits against protein kinase Cζ (PKCζ) (sc-216, SantaCruz Biotechnology, Inc.), INSR and PI3K (for all: diluted at 1:200 in 5% skimmed milk-PBST) for adipose tissues, at 4°C overnight. After three wash cycles in PBST of 5 min, membranes were incubated with the secondary antibody, horse-radish peroxidase-conjugated mouse anti-rabbit IgG antibody diluted in 2.5% skimmed milk-PBST to 1:2500 for detecting hepatic GLUT2, hepatic PI3K and to 1:12500 for detecting hepatic INSR at room temperature for 120 min. For detecting the investigated proteins in adipose tissue the secondary antibody was diluted in 5% skimmed milk-PBST to 1:2500. Membranes were then washed three times for 5 min with PBST and once for 10 min with PBS and incubated with LumiGLO substrate (Kirkegaard & Perry Laboratories, Inc., Gaithersburg, USA). Detecting and recording chemiluminescence signals of membranes were performed using a Molecular Imager ChemiDoc XRS+ System (Bio-Rad Laboratories, Inc.). Membranes were rinsed with distilled water and stained with India ink to confirm equal loading. The analysis of each tissue sample was repeated three times for each cow. Specificity of primary antibody was confirmed using antibody-specific blocking peptides for the antibodies against GLUT2, INSR, and PKCζ. For PI3K, no specific peptide was available, but an isotype control serum was used for validation. Primary antibodies were incubated with or without five times higher amount of blocking peptide in 2.5% skimmed milk-PBST at room temperature for 2 h and then was applied on membrane. Omitting the specific signal by blocking with the respective antigenic peptide was considered to confirm specificity of heterologous antibodies to detect marker proteins in bovine tissues. Representative signals of the investigated proteins and the results of the specificity test are presented as supporting information (S1 Fig).

### Data processing and statistical analysis

Signal intensity of bands was measured densitometrically using Image Lab Software (Bio-Rad Laboratories, Inc.). By calculating the ratio of the protein quantities (d21/d-21), the changes in protein expression from day -21 to day 21 were determined. A ratio > 1 indicates that the expression of the protein at d21 was higher and a ratio < 1 indicates that it was lower than that at d-21. Thus, the ratio represented the dynamic changes of protein expression between the two time points. Since this method enabled to normalize variations between membranes as well as between individual cows at once, no further statistical modeling regarding the random factors was needed. The statistical analyses were performed using SAS (Version 9.2, SAS Institute Inc., Cary, NC, USA). The ratios of protein expression were analyzed using general linear model (PROC GLM) for the fixed factors dietary NA supplementation (Na) and concentrate proportion (Conc), and their interaction. Moreover, one sample t-test was performed using all the data (n = 20) to test whether the ratios differed significantly from 1, in other words, whether there were time (onset of lactation) effects. The blood metabolites, plasma nicotinamide concentration, feed intake and milk performance were analyzed for the effects of Na, Conc and time and their interactions using mixed model with repeated measures (PROC MIXED) [18] with “cow” as a random factor. Restricted maximum likelihood was used for estimation of means and Kenward-Roger degrees of freedom approximation was used for the test of fixed effects. The best fit covariance structures were decided from compound symmetry, component variance, and unstructured using the Akaike information criterion (AIC) for each variable. The values at the first sampling day (d-21) in each evaluation data set were integrated as covariables in the mixed model. The normality of distribution of residues was checked by Shapiro-Wilk test (PROC UNIVARIATE). In case of that the fixed effects were significant in variance analyses, the differences of estimated group means were tested using t-test. Levels of significance and of relevant trend were set at p < 0.05 and 0.1, respectively.

## Results

### Feed, nutrient intake and performance and plasma nicotinamide concentration

Results of feed and nutrient intake and performance after calving and plasma nicotinamide concentration at d21 are shown in Table 1. Intake of dry matter and net energy lactation (NEL) were higher in groups fed a high concentrate proportion (HC-CON and HC-NA) and showed an increase in all groups. Intake of silages was higher and increased mainly in groups fed a low concentrate proportion. Higher and increasing intake of concentrates was observed mainly in groups fed a high concentrate proportion. However, the intake of concentrate did not reach the intended amount of 60% concentrate on a DM basis in groups with a high concentrate proportion. Milk yields were higher in groups fed a high concentrate proportion. The mean plasma nicotinamide concentration was higher in groups fed NA than in the control, as expected. The plasma concentration of NA was below the detection limit.

**Table 1:** Dry matter intake, energy intake, milk yield, and plasma nicotinamide concentration at day 21 (d21) related to calving.

	Group mean^a^					Probability of fixed effects in type 3 test^b^					
Variable	LC-CON	HC-CON	LC-NA	HC-NA	Pooled SEM	Time	Na	Conc	Na × Conc	Na × time	Conc × time
Dry matter intake, kg/day	14.7	17.6	14.7	16.4	0.51	<0.001	0.28	<0.001	0.22	0.96	0.72
Maize silage intake, kg DM/day	6.25	5.34	6.34	5.11	0.26	0.18	0.77	<0.001	0.52	0.80	0.015
Gras silage intake, kg DM/day	4.17	3.57	4.26	3.43	0.17	0.18	0.88	<0.001	0.50	0.81	0.017
Concentrate intake, kg DM/day	4.24	8.71	4.13	7.90	0.28	<0.001	0.11	<0.001	0.22	0.86	<0.001
NEL intake, MJ/day	98.3	123.9	99.3	117.1	3.82	<0.001	0.45	<0.001	0.32	0.97	0.45
Milk yield, kg/day	29.9	34.9	27.5	34.1	1.90	0.11	0.41	0.004	0.69	0.67	0.95
Nicotinamide in plasma at d21, μg/mL	0.66	0.66	1.40	0.92	0.13	-	0.001	0.08	0.08	-	-

^a^ Group mean: mean from d1 to d21 after calving in each group (n = 5 cows/group); LC-CON: low concentrates (30% on dry matter basis) diet from d1 to d21; HC-CON: high concentrates (60% on dry matter basis) from d1 to d21; LC-NA: low concentrate + 24 g/d nicotinic acid from d1 to d21; HC-NA: high concentrates + 24 g/d nicotinic acid from d1 to d21, SEM: standard error of the mean.

^b^ Na: effect of nicotinic acid supplementation; Conc: effect of concentrate proportion; Na × Conc, Na × time, Conc × time: interaction effects between time, Na, and Conc, DM: dry matter, NEL: net energy lactation.

### Glucose, NEFA, insulin, and RQUICKI

Results of the analysis of blood metabolites and RQUICKI at d-21 and at d21 are shown in Table 2. Glucose concentration decreased significantly over time in control groups but not in NA-fed groups. In the other metabolites and RQUICKI, only time effects were found. Insulin concentration decreased over time. Similarly, a trend of decrease over time (*P* = 0.057) was found in RQUICKI. As expected, NEFA concentration were higher post partum.

**Table 2:** Concentration of glucose, NEFA, insulin, and RQUICKI at day -21 (d-21) and day 21 (d21) related to calving.

		Group mean^b^					Probability of fixed effects in type 3 test^c^					
Variable^a^		LC-CON	HC-CON	LC-NA	HC-NA	SEM	Time	Na	Conc	Na × Conc	Na × time	Conc × time
Glucose, m*M*	d-21	3.87	3.90	3.85	3.94	0.073	<0.001	0.002	0.48	0.99	0.003	0.69
	d21	3.48	3.53	3.85	3.84							
Insulin, μU/mL	d-21	17.3	17.6	20.4	21.6	3.18	0.004	0.27	0.50	0.81	0.69	0.74
	d21	10.5	12.2	11.7	14.5							
Log NEFA^d^, μ*M*	d-21	2.44	2.38	2.42	2.36	0.12	<0.001	0.75	0.46	0.91	0.91	0.98
	d21	2.82	2.77	2.80	2.72							
RQUICKI	d-21	0.413	0.414	0.407	0.412	0.017	0.057	0.39	0.68	0.76	0.59	0.49
	d21	0.406	0.387	0.383	0.374							

^a^ NEFA: nonesterified fatty acids, RQUICKI: revised insulin sensitivity check index.

^b^ n = 5 cows/group; LC-CON: low concentrate (30% on dry matter basis) from d1 to d21; HC-CON = high concentrates (60% on dry matter basis) from d1 to d21; LC-NA: low concentrates + 24 g/d nicotinic acid from d1 to d21; HC-NA: high concentrates + 24 g/d nicotinic acid from d1 to d21, SEM: standard error of the mean

^c^ Na: effect of nicotinic acid supplementation; Conc: effect of concentrate proportion; Na × Conc, Na × time, Conc × time: interaction effects between time, Na, and Conc.

^d^ values after logarithmic transformation.

### Expression of INSR, PI3K, GLUT2, and PKCζ proteins

Representative signals of Western Blot analysis are shown in S1 Fig. Signals for proteins of interest were detected at expected molecular weight, 95 kDa for INSR, 85 kDa for PI3K, 60 kDa for GLUT2 and 80 and 60 kDa for PKCζ, in each organ, respectively. These signals were omitted when samples were treated with antibodies which were pre-incubated with the specific blocking peptide. In the liver anti-insulin receptor antibodies detected two specific signals at about 200 kDa as the precursor of INSR and 95 kDa as the mature form of INSR, whereas in SCAT and RPAT only the mature receptor at 95 kDa was detected. The double band for PKCζ cannot be explained yet, but both of them were assessed as being specific.

### Effects of time and diet on hepatic protein expression

**Fig 1:**
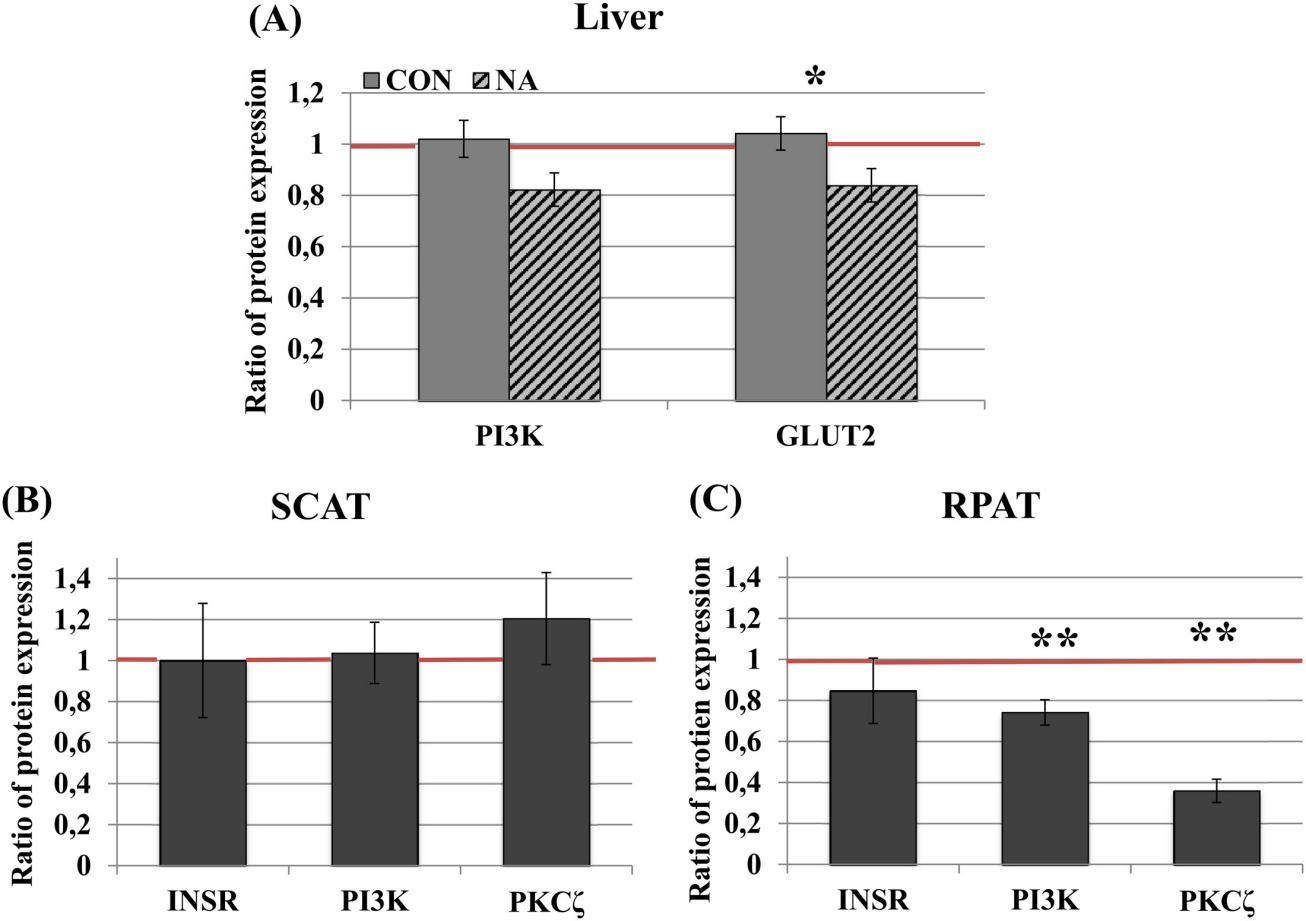
Hepatic protein expression as affected by NA and adipose protein expression as affected by time. A: The ratios of pooled groups (pool “NA” = HC-NA, LC-NA and pool “CON” = HC-CON, LC-CON, 24 or 0 g/d nicotinic acid from d1 to d21) were tested for the influence of nicotinic acid supplementation on the hepatic protein expression of PI3K and GLUT2 by one-way ANOVA (n = 10/group). Hepatic GLUT2 expression decreased from d-21 to d21 (ratio < 1) in cows fed NA, and it was lower in cows fed NA (*p = 0.03) compared to control cows at d21. B, C: The ratios of the protein expression in subcutaneous (B, “SCAT”) and retroperitoneal (C, “RPAT”) adipose tissue from all the cows. Cows were pooled (HC-NA, LC-NA, HC-CON, LC-CON) and tested for the difference to 1 by one-sample t-test (n = 20). The expression of signaling proteins in RPAT was generally lower at d21 compared to at d-21 (ratio < 1; **p < 0.01). Ratio = protein expression d21/d-21; ratio > 1: increase, ratio < 1: decrease; The red line indicates the ratio of protein expression at d-21 (d-21/d-21 = 1). Data are shown as mean ± standard error. NA: nicotinic acid, PI3K: phosphatidylinositol-3-kinase, GLUT2: glucose transporter 2, INSR: insulin receptor, PKCζ = protein kinase Cζ.

The mean ratio calculated from protein expression of all the investigated cows were not significantly different from 1 for all the measured proteins (for INSR, precursor, PI3K, GLUT2 ratios: 0.95 ± 0.05, 0.98 ± 0.07, 0.92 ± 0.06, 0.94 ± 0.05, mean ± standard error, n = 20). Results for diet effects on hepatic protein expression are shown in Table 3. For INSR and its precursor no diet effects were found. In PI3K (*P* = 0.08) and GLUT2 (*P* = 0.03), NA effects were observed with lower ratios of protein expression in groups fed NA (Fig 1).

**Table 3:** Effects of diet on the ratio of the expression^a^ of hepatic key proteins of insulin signaling and glucose transport.

	Group mean^c^					Probability of fixed effects in type 3 test^d^		
Variable^b^	LC-CON	HC-CON	LC-NA	HC-NA	PooledSEM	Na	Conc	Na × Conc
INSR	1.01	1.01	0.96	0.80	0.09	0.17	0.40	0.37
Precursor	1.04	1.11	0.87	0.91	0.15	0.19	0.71	0.91
PI3K	1.00	1.04	0.87	0.77	0.11	0.082	0.76	0.53
GLUT2	0.918	1.17	0.81	0.87	0.087	0.033	0.090	0.31

^a^ ratio = protein expression d21/d-21; ratio > 1: increase, ratio < 1: decrease.

^b^ INSR: insulin receptor, precursor: precursor of INSR; PI3K: phosphatidylinositol-3-kinase; GLUT2: glucose transporter 2.

^c^ n = 5 cows/group; LC-CON: low concentrate (30% on dry matter basis) from d1 to d21; HC-CON: high concentrate (60% on dry matter basis) from d1 to d21; LC-NA: low concentrate + 24 g/d nicotinic acid from d1 to d21; HC-NA: high concentrate + 24 g/d nicotinic acid from d1 to d21; SEM: standard error of the mean.

^d^ Na: effect of nicotinic acid supplementation; Conc: effect of concentrate proportion; Na × Conc: interaction effects between Na and Conc.

### Effects of time and diet on protein expression in SCAT and RPAT

In case of of PI3K and PKCζ in RPAT, the ratios calculated from the protein expression were significanty lower than 1, indicating lower protein expression at d-21 compared to d21. On the contrary, no time effect was found in SCAT (Fig 1). No diet effects were found in investigated proteins in SCAT and RPAT (Table 4).

**Table 4:** Effects of diet on the ratio of the expression^a^ of adipose key proteins of insulin signaling.

	Group mean^c^					Probability of fixed effects in type 3 test^d^		
Variable^b^	LC-CON	HC-CON	LC-NA	HC-NA	PooledSEM	Na	Conc	Na × Conc
*SCAT*								
INSR	0.78	2.01	0.57	0.60	0.51	0.13	0.23	0.27
PI3K	0.94	1.32	0.96	0.93	0.31	0.56	0.57	0.52
PKCζ	1.37	1.30	1.22	0.86	0.47	0.54	0.66	0.76
*RPAT*								
INSR	0.64	0.86	1.19	0.66	0.32	0.59	0.64	0.26
PI3K	0.82	0.71	0.68	0.90	0.14	0.87	0.71	0.28
PKCζ	0.27	0.29	0.48	0.58	0.14	0.10	0.69	0.79

^a^ ratio = protein expression d21/d-21; ratio > 1: increase, ratio < 1: decrease.

^b^ SCAT = subcutaneous adipose tissue; RPAT = retroperitoneal adipose tissue; INSR = insulin receptor; PI3K = phosphatidylinositol-3-kinase; PKCζ = protein kinase Cζ.

^c^ n = 5 cows/group; LC-CON: low concentrate (30% on dry matter basis) from d1 to d21; HC-CON: high concentrate (60% on dry matter basis) from d1 to d21; LC-NA: low concentrate + 24 g/d nicotinic acid from d1 to d21; HC-NA: high concentrate + 24 g/d nicotinic acid from d1 to d21; SEM: standard error of the mean.

^d^ Na: effect of nicotinic acid supplementation; Conc: effect of concentrate proportion; Na × Conc: interaction effects between Na and Conc.

## Discussion

The present study investigated the effects of postpartum diet differing in concentrate proportion and NA supplementation on the dynamic changes of expression of proteins involved in insulin signaling and glucose metabolism in the liver and adipose tissue in dairy cows in the periparturient period. However, the effect of concentrate proportion in this study should be considered as an effect of dietary energy intake, because the ingested amount of concentrate in HC-CON and HC-NA did not reach 60% in dry matter basis.

The main findings in this study were 1) the marked reduction of PI3K and PKCζ protein expression over time in RPAT, and 2) the lower content of hepatic GLUT2 protein in cows supplemented with NA.

### Effects of time and diet on insulin signaling in adipose tissues and liver

In the investigated insulin signaling molecules in the liver and adipose tissues, marked time effects were observed in RPAT only. The decrease in expression of PI3K and PKCζ indicated an attenuated capacity of RPAT to respond to insulin. This may be interpreted as a reduction in tissue-specific insulin sensitivity. However, the physiological role of this local effect in RPAT for systemic insulin sensitivity is unclear. A tissue-specific regulation of insulin responsiveness in cattle was also discussed by McGrattan et al. [19] who demonstrated that insulin receptor concentration in subcutaneous adipose tissue was lower than that in omental adipose tissue in steers.

In SCAT and the liver, none of the investigated key proteins in insulin signaling were influenced by time. However, according to Ji et al., the mRNA expression of INSR and other insulin signaling molecules and the extent of phosphorylation of tyrosine residues of insulin receotor substrate 1 in SCAT decreased at onset of lactation in dairy cows [20]. These differences in results could indicate that the regulation of insulin signaling takes place at transcriptional, translational and post translational levels.

Insulin binds to insulin receptor and activates the receptor-own tyrosine kinase, which leads to activation of PI3K [21]. This results in an increase in the amount of phosphatidylinositol-3-phosphate, activating PKCζ. The activation of PKCζ is required for the translocation of glucose transporter 4 (GLUT4) into the plasma membrane and thereby, for the subsequent cellular glucose uptake [22]. In adipose tissue, re-esterification of fatty acids takes place parallel to lipolysis, and glycerol required for re-esterification can be supplied only from de novo synthesis from glucose [1]. Therefore one of the possible physiological consequences of the reduced expression of PI3K and PKCζ in RPAT might be a decreased fatty acid re-esterification. Confirming potential metabolic differences of RPAT and SCAT, Locher et al. [16] found that hormone-sensitive lipase (HSL) phosphorylation at serine 660 as an indicator for lipolytic activity, increased significantly at d21 in RPAT, but not in SCAT. According to von Soosten et al. [23], a significant decrease in weight was seen in RPAT, but not SCAT at d42 compared to d-21 in dairy cows. These could indicate higher lipolytic activity and lower activity of re-esterification in RPAT compared to in SCAT. However, postpartal decrease of re-esterification also occurred in SCAT [24].

In this study, no clear evidence was found regarding the dietary effect on hepatic and adipose insulin signaling. So far, influences of NA on insulin sensitivity have been investigated in dairy cows only in vivo [7,25,26]. Any potential direct effect of NA on expression of hepatic PI3K and INSR in dairy cows is still unknown. In rats, NA-induced reduction of expression of genes involved in hepatic insulin signaling was reported [27].

### Effects of time on hepatic GLUT2 expression

As hepatic glucose output increased drastically at the onset of lactation in dairy cows [2], time-related increase of GLUT2 was expected. However, the hepatic GLUT2 protein expression was not affected by time. Possible explanations for the lack of time-related changes in GLUT2 expression are: 1) that regulation on hepatic GLUT2 expression in dairy cows are independent of insulin sensitivity as well as a lactation period and 2) that the insulin-dependent internalization of GLUT2 in hepatocytes (reviewed by Karim et al.) [28] had a greater impact than the regulation on the expression levels. Assuming the latter explanation is correct, an increased number of functional GLUT2 might be translocated on the surface of hepatocytes after calving without changes to the total protein amount. However, the mechanism is unknown.

### Possible mechanism of NA effects on protein expression

In the present study, effects of NA supplementation on key protein expression were detected in the liver, but not in adipose tissues. However, the impact and physiological role of NA effects on liver remain unclear. The reason for the tissue-specific differences of dietary effects may be based on NA kinetics. The NA absorbed from the gastrointestinal tract is converted into NAD in the liver and released in form of nicotinamide, while a minor part of NA will leave the liver without being converted [8]. Thus, NA effects are most likely occurring only in the liver.

For the mechanisms of NA actions on gene expression several direct and indirect pathways were suggested. NA might affect gene expression indirectly by modifying the concentration of hormones and blood metabolites [5]. One of the direct pathways seemed to be the interaction with the NA receptor (GRP109A). It was suggested that the activation of GPR109 and downstream signal cascade could lead to activation or inactivation of transcriptional factors [5]. In cattle, a substantial amount of GPR109A expression was found in the liver [29]. Therefore it might be likely that the observed hepatic NA effects were caused by the direct effects of NA through hepatic GPR109A. The absence of NA effects on adipose tissues regarding antilipolysis and insulin sensitivity might be explained by the fact that only NA, but not nicotinamide acted as an agonist of GPR109A [6,30]. Consequently, only GLUT2 in the liver was affected by feeding without any concomitant effects on serum NEFA concentration. Previous studies demonstrated that NA modified expression of several genes involved in energy metabolism in pigs (e.g. PGC-1β; peroxisome proliferator-activated receptor gamma, coactivator 1 beta, skeletal muscle) [31], in sheep (e.g. SDHA; succinate dehydrogenase complex, subunit A, skeletal muscle) [32], and in rats (e.g. GLUT2, liver) [27].

## Conclusions

Modulation of insulin signaling at the onset of lactation occurred at the level of protein expression in a tissue-specific manner irrespectivley of diet. Dietary NA supplementation was able to reduce the expression of hepatic GLUT2 protein; however, the physiological importance is unclear. Lack of strongly significant effects of NA supplementation on serum NEFA concentrations, lipid and glucose metabolism in the liver and adipose tissue indicated that feeding NA may not be highly efficient to improve metabolic health of periparturient dairy cows.

## Supporting Information

S1 Fig Representative signals of investigated proteins. A: GLUT2, B: INSR, C: PI3K, D: PKCζ; A–D: Representative signals of investigated proteins in the liver (at d-21 and d21, A–C) and RPAT (at d21, D) of cows from each experimental group; A1 –D1: Representative signals of investigated proteins in the liver (A–C) and RPAT (D); A2 –D2: Specificity test.
Reduced and denatured protein of hepatic samples (40 μg) and protein from RPAT samples (20 μg) were loaded onto PAGE gels and then transferred to nitrocellulose membrane. Membranes were blocked with 5% skimmed milk-PBST (GLUT2, INSR in the liver and PI3K and PKCζ in RPAT) or 5% BSA-PBST (PI3K in the liver) and incubated with primary antibodies diluted at 1:100 (GLUT2 in the liver) or at 1:400 (INSR in the liver) in 2.5% skimmed milk-PBST or at 1:200 in 2.5% BSA-PBST (PI3K in the liver) or at 1:200 in 5% skimmed milk-PBST (PI3K and PKCζ in RPAT) at 4°C overnight, and with secondary antibodies diluted at 1:5000 (GLUT2 and PI3K in the liver) or at 1:50000 (INSR in the liver) or at 1:2500 (PI3K and PKCζ in RPAT) at room temperature for 2 h. For specificity-test, antibodies were incubated with 5 times greater amounts of blocking peptide (“Blocked” in A2, B2, D2) or isotype control antibody („Isotype”in C2) at room temperature for 2 h and applied to a representative membrane. India ink stain are presented as internal controls. RPAT: reptroperitoneal adipose tissue, LC-CON, HC-CON, LC-NA, HC-NA: “CON or NA”: dietary supplement of nicotinic acid (0 or 24 g/d) from the day after calving to d21, “LC or HC”: 30 or 60% of concentrate proportion in the diet from the day after calving to d21, control: control samples for inter membrane controls, d: days in milk, GLUT2: glucose transporter 2, INSR: insulin receptor, PI3K: phosphatidylinositol-3-kinase, PKCζ: protein kinase Cζ.
(TIFF)

S1 Table Nutrient and energy content of silages and concentrates. ^a^ LC-CON, HC-CON, LC-NA, HC-NA: “CON or NA”: dietary supplement of nicotinic acid (0 or 24 g/d) from the day after calving to d21, “LC or HC”: 30 or 60% of concentrate proportion in the diet from the day after calving to d21, control: control samples for inter membrane controls. ^b^ Per kg mineral feed: 140 g Ca; 120 g Na; 70 g P; 40 g Mg; 6 g Zn; 5.4 g Mn; 1 g Cu; 100mg I; 40 mg Se; 5 mg Co; 1 000 000 IU vitamin A; 100 000 IU vitamin D3; 1500 mg vitamin E. ^c^ Calculation based on nutrient digestibilities measured in wethers [33]. ^d^ Calculation based on analyzed nutrient contents and tabulated values of apparent digestibilities [34].
(PDF)

## References

[1] Herdt TH. Ruminant adaptation to negative energy balance. Influences on the etiology of ketosis and fatty liver. Vet Clin North Am Food Anim Pract. 2000;16: 215–230, v. 11022337 10.1016/s0749-0720(15)30102-x

[2] Bell AW, Bauman DE. Adaptations of glucose metabolism during pregnancy and lactation. J Mammary Gland Biol Neoplasia. 1997;2: 265–278. 10882310 10.1023/a:1026336505343

[3] Duehlmeier R, Sammet K, Widdel A, von Engelhardt W, Wernery U, Kinne J, et al Distribution patterns of the glucose transporters GLUT4 and GLUT1 in skeletal muscles of rats (Rattus norvegicus), pigs (Sus scrofa), cows (Bos taurus), adult goats, goat kids (Capra hircus), and camels (Camelus dromedarius). Comp Biochem Physiol A Mol Integr Physiol. 2007;146: 274–282. 10.1016/j.cbpa.2006.10.029 17158080

[4] Komatsu T, Itoh F, Kushibiki S, Hodate K. Changes in gene expression of glucose transporters in lactating and nonlactating cows. J Anim Sci. 2005;83: 557–564. 15705752 10.2527/2005.833557x

[5] Kang I, Kim S-W, Youn JH. Effects of nicotinic acid on gene expression: potential mechanisms and implications for wanted and unwanted effects of the lipid-lowering drug. J Clin Endocrinol Metab. 2011;96: 3048–3055. 10.1210/jc.2011-1104 21816780 PMC3200242

[6] Kenéz A, Locher L, Rehage J, Dänicke S, Huber K. Agonists of the G protein-coupled receptor 109A-mediated pathway promote antilipolysis by reducing serine residue 563 phosphorylation of hormone-sensitive lipase in bovine adipose tissue explants. J Dairy Sci. 2014;97: 3626–34. 10.3168/jds.2013-7662 24704242

[7] Pires JAA, Pescara JB, Grummer RR. Reduction of plasma NEFA concentration by nicotinic acid enhances the response to insulin in feed-restricted Holstein cows. J Dairy Sci. 2007;90: 4635–4642. 10.3168/jds.2007-0146 17881684

[8] Niehoff I-D, Hüther L, Lebzien P. Niacin for dairy cattle: a review. Br J Nutr. 2009;101: 5–19. 10.1017/S0007114508043377 18702847

[9] Rauls C, Meyer U, Hüther L, von Soosten D, Kinoshita A, Rehage J, et al Effects of niacin supplementation (40 weeks) and two dietary levels of concentrate on performance, blood and fatty acid profiles of dairy cattle. 2015;45: 395–410.

[10] Nordlie RC, Foster JD, Lange AJ. Regulation of glucose production by the liver. Annu Rev Nutr. 1999;19: 379–406. 10.1146/annurev.nutr.19.1.379 10448530

[11] Zhao F-Q, Kennelly JJ, Moseley WM, Tucker HA. Regulation of the Gene Expression of Glucose Transporter in Liver and Kidney of Lactating Cows by Bovine Growth Hormone and Bovine Growth Hormone-Releasing Factor. J Dairy Sci. 1996;79: 1537–1542. 10.3168/jds.S0022-0302(96)76514-1 8899518

[12] Hammon HM, Metges CC, Schulz A, Junghans P, Steinhoff J, Schneider F, et al Differences in milk production, glucose metabolism, and carcass composition of 2 Charolais × Holstein F2 families derived from reciprocal paternal and maternal grandsire crosses1. J Dairy Sci. 2010;93: 3007–3018. 10.3168/jds.2009-2931 20630217

[13] VDLUFA (Verband Deutscher Landwirtschaftlicher Untersuchungs- und Forschungsanstalten), editor. VDLUFA-Methodenbuch Band III: Die chemische Untersuchung von Futtermitteln Erganzungslieferungen von 1983, 1988, 1992, 1997, 2004, 2006, 2007. Darmstadt: VDLUFA-Verl; 1997.

[14] Holtenius P, Holtenius K. A model to estimate insulin sensitivity in dairy cows. Acta Vet Scand. 2007;49: 29 10.1186/1751-0147-49-29 17931417 PMC2092429

[15] Niehoff I-D, Huther L, Lebzien P, Bigalke W, Danicke S, Flachowsky G. Investigations on the effect of a niacin supplementation to three diets differing in forage-to-concentrate ratio on several blood and milk variables of dairy cows. Arch Anim Nutr. 2009;63: 203–218. 10.1080/17450390902863764

[16] Locher LF, Meyer N, Weber E-M, Rehage J, Meyer U, Dänicke S, et al Hormone-sensitive lipase protein expression and extent of phosphorylation in subcutaneous and retroperitoneal adipose tissues in the periparturient dairy cow. J Dairy Sci. 2011;94: 4514–4523. 10.3168/jds.2011-4145 21854923

[17] Laemmli UK. Cleavage of structural proteins during the assembly of the head of bacteriophage T4. Nature. 1970;227: 680–685. 5432063 10.1038/227680a0

[18] Littell RC, Henry PR, Ammerman CB. Statistical analysis of repeated measures data using SAS procedures. J Anim Sci. 1998;76: 1216–1231. 9581947 10.2527/1998.7641216x

[19] McGrattan PD, Wylie ARG, Nelson J. Tissue-specific differences in insulin binding affinity and insulin receptor concentrations in skeletal muscles, adipose tissue depots and liver of cattle and sheep. Anim Sci. 2000;71: 501–508.

[20] Ji P, Osorio JS, Drackley JK, Loor JJ. Overfeeding a moderate energy diet prepartum does not impair bovine subcutaneous adipose tissue insulin signal transduction and induces marked changes in peripartal gene network expression. J Dairy Sci. 2012;95: 4333–4351. 10.3168/jds.2011-5079 22818447

[21] Hayirli A. The role of exogenous insulin in the complex of hepatic lipidosis and ketosis associated with insulin resistance phenomenon in postpartum dairy cattle. Vet Res Commun. 2006;30: 749–774. 10.1007/s11259-006-3320-6 17004039

[22] Farese RV, Sajan MP. Metabolic functions of atypical protein kinase C: “good” and “bad” as defined by nutritional status. Am J Physiol Endocrinol Metab. 2010;298: E385–394. 10.1152/ajpendo.00608.2009 19996389 PMC3774273

[23] von Soosten D, Meyer U, Weber EM, Rehage J, Flachowsky G, Dänicke S. Effect of trans-10, cis-12 conjugated linoleic acid on performance, adipose depot weights, and liver weight in early-lactation dairy cows. J Dairy Sci. 2011;94: 2859–2870. 10.3168/jds.2010-3851 21605756

[24] Rukkwamsuk T, Wensing T, Geelen MJ. Effect of overfeeding during the dry period on the rate of esterification in adipose tissue of dairy cows during the periparturient period. J Dairy Sci. 1999;82: 1164–1169. 10.3168/jds.S0022-0302(99)75339-7 10386302

[25] Chilliard Y, Ottou JF. Duodenal infusion of oil in midlactation cows. 7. Interaction with niacin on responses to glucose, insulin, and beta-agonist challenges. J Dairy Sci. 1995;78: 2452–2463. 10.3168/jds.S0022-0302(95)76873-4 8747336

[26] Lanham JK, Coppock CE, Brooks KN, Wilks DL, Horner JL. Effects of whole cottonseed or niacin or both on casein synthesis by lactating Holstein cows. J Dairy Sci. 1992;75: 184–192. 10.3168/jds.S0022-0302(92)77752-2 1311726

[27] Choi S, Yoon H, Oh K-S, Oh YT, Kim YI, Kang I, et al Widespread effects of nicotinic acid on gene expression in insulin-sensitive tissues: implications for unwanted effects of nicotinic acid treatment. Metabolism. 2011;60: 134–144. 10.1016/j.metabol.2010.02.013 20303128 PMC2912158

[28] Karim S, Adams DH, Lalor PF. Hepatic expression and cellular distribution of the glucose transporter family. World J Gastroenterol WJG. 2012;18: 6771–6781. 10.3748/wjg.v18.i46.6771 23239915 PMC3520166

[29] Titgemeyer EC, Mamedova LK, Spivey KS, Farney JK, Bradford BJ. An unusual distribution of the niacin receptor in cattle. J Dairy Sci. 2011;94: 4962–4967. 10.3168/jds.2011-4193 21943747

[30] Wise A, Foord SM, Fraser NJ, Barnes AA, Elshourbagy N, Eilert M, et al Molecular identification of high and low affinity receptors for nicotinic acid. J Biol Chem. 2003;278: 9869–9874. 10.1074/jbc.M210695200 12522134

[31] Khan M, Ringseis R, Mooren F-C, Krüger K, Most E, Eder K. Niacin supplementation increases the number of oxidative type I fibers in skeletal muscle of growing pigs. BMC Vet Res. 2013;9: 177 10.1186/1746-6148-9-177 24010567 PMC3846775

[32] Khan M, Couturier A, Kubens JF, Most E, Mooren F-C, Krüger K, et al Niacin supplementation induces type II to type I muscle fiber transition in skeletal muscle of sheep. Acta Vet Scand. 2013;55: 85 10.1186/1751-0147-55-85 24267720 PMC4176759

[33] Leitlinien für die Bestimmung der Verdaulichkeit von Rohnährstoffen an Wiederkäuern. J Anim Physiol Anim Nutr. 1991;65: 229–234. 10.1111/j.1439-0396.1991.tb00261.x

[34] DLG. DLG Futterwerttabellen Wiederkäuer (Feed value tables for ruminants). 7th ed Frankfurt am Main: DLG-Verlag; 1997.

