# Supplementary figures and images for: Insulin Signaling in Liver and Adipose Tissues in Periparturient Dairy Cows Supplemented with Dietary Nicotinic Acid
Source: PLoS One. 2016 Jan 14;11(1):e0147028. doi: 10.1371/journal.pone.0147028 (PMC4713095; doi:10.1371/journal.pone.0147028)

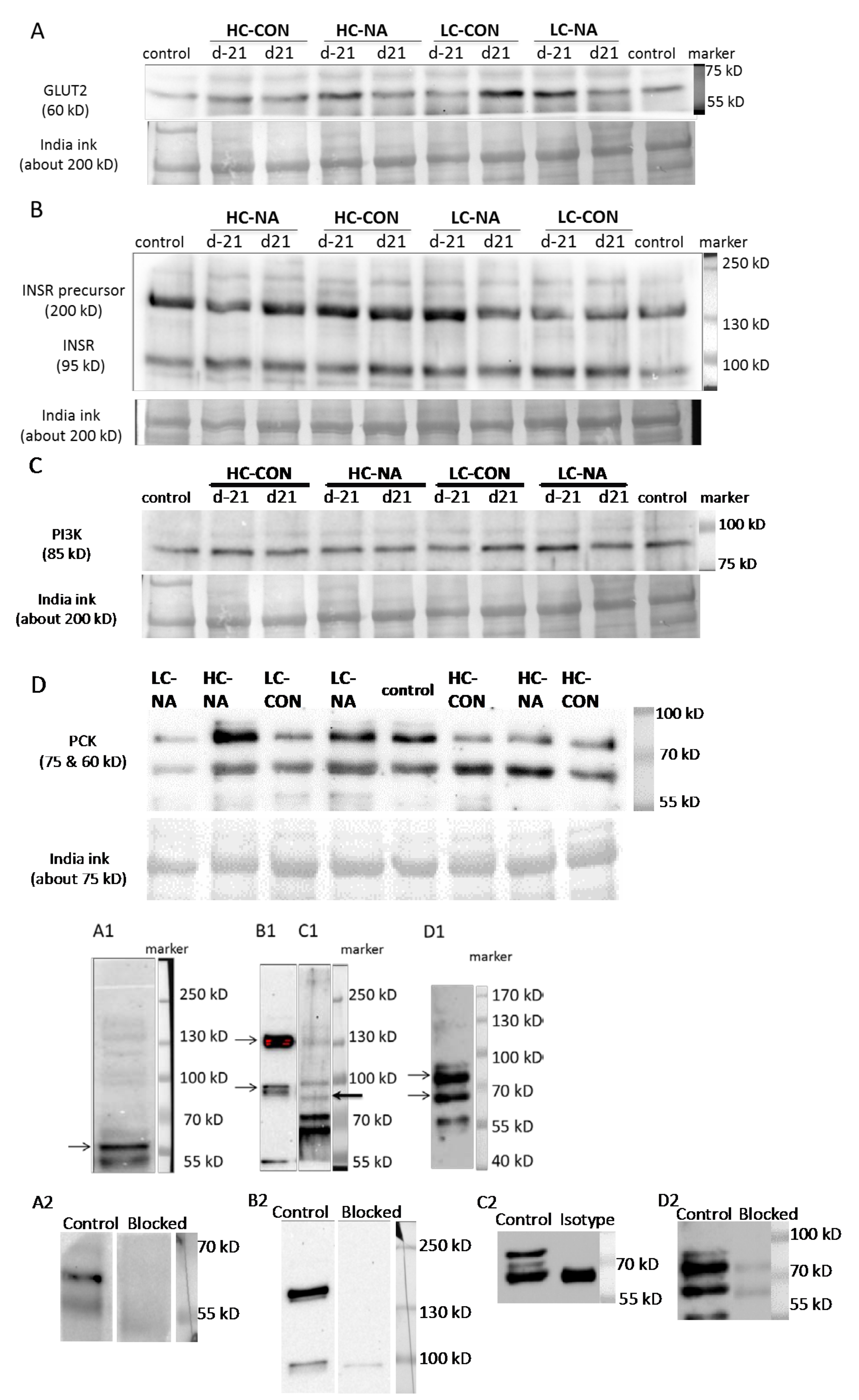

Supplement: S1 Fig — A: GLUT2, B: INSR, C: PI3K, D: PKCζ; A–D: Representative signals of investigated proteins in the liver (at d-21 and d21, A–C) and RPAT (at d21, D) of cows from each experimental group; A1 –D1: Representative signals of investigated proteins in the liver (A–C) and RPAT (D); A2 –D2: Specificity test. Reduced and denatured protein of hepatic samples (40 μg) and protein from RPAT samples (20 μg) were loaded onto PAGE gels and then transferred to nitrocellulose membrane. Membranes were blocked with 5% skimmed milk-PBST (GLUT2, INSR in the liver and PI3K and PKCζ in RPAT) or 5% BSA-PBST (PI3K in the liver) and incubated with primary antibodies diluted at 1:100 (GLUT2 in the liver) or at 1:400 (INSR in the liver) in 2.5% skimmed milk-PBST or at 1:200 in 2.5% BSA-PBST (PI3K in the liver) or at 1:200 in 5% skimmed milk-PBST (PI3K and PKCζ in RPAT) at 4°C overnight, and with secondary antibodies diluted at 1:5000 (GLUT2 and PI3K in the liver) or at 1:50000 (INSR in the liver) or at 1:2500 (PI3K and PKCζ in RPAT) at room temperature for 2 h. For specificity-test, antibodies were incubated with 5 times greater amounts of blocking peptide (“Blocked” in A2, B2, D2) or isotype control antibody („Isotype”in C2) at room temperature for 2 h and applied to a representative membrane. India ink stain are presented as internal controls. RPAT: reptroperitoneal adipose tissue, LC-CON, HC-CON, LC-NA, HC-NA: “CON or NA”: dietary supplement of nicotinic acid (0 or 24 g/d) from the day after calving to d21, “LC or HC”: 30 or 60% of concentrate proportion in the diet from the day after calving to d21, control: control samples for inter membrane controls, d: days in milk, GLUT2: glucose transporter 2, INSR: insulin receptor, PI3K: phosphatidylinositol-3-kinase, PKCζ: protein kinase Cζ. (TIFF) [file pone.0147028.s001.tiff]
