# Supplementary material for: Insulin Signaling in Liver and Adipose Tissues in Periparturient Dairy Cows Supplemented with Dietary Nicotinic Acid
Source: PLoS One. 2016 Jan 14;11(1):e0147028. doi: 10.1371/journal.pone.0147028 (PMC4713095; doi:10.1371/journal.pone.0147028)
Supplement: S1 Table — a LC-CON, HC-CON, LC-NA, HC-NA: “CON or NA”: dietary supplement of nicotinic acid (0 or 24 g/d) from the day after calving to d21, “LC or HC”: 30 or 60% of concentrate proportion in the diet from the day after calving to d21, control: control samples for inter membrane controls. b Per kg mineral feed: 140 g Ca; 120 g Na; 70 g P; 40 g Mg; 6 g Zn; 5.4 g Mn; 1 g Cu; 100mg I; 40 mg Se; 5 mg Co; 1 000 000 IU vitamin A; 100 000 IU vitamin D3; 1500 mg vitamin E. c Calculation based on nutrient digestibilities measured in wethers [33]. d Calculation based on analyzed nutrient contents and tabulated values of apparent digestibilities [34]. (PDF) [file pone.0147028.s002.pdf]

**S1 Table. Nutrient and energy content of silages and concentrates**

| | | | Concentrate[a] | | | |
|---|---|---|---|---|---|---|
| | Maize silage | Grass silage | LC-CON | HC-CON | LC-NA | HC-NA |
| *Components (%)* | | | | | | |
| Wheat grain | | | 50 | 50 | 50 | 50 |
| Maize | | | 20 | 20.8 | 20 | 20.8 |
| Soybean meal | | | 26 | 26.8 | 26 | 26.8 |
| Mineral premix[b] | | | 4 | 2.4 | 4 | 2.4 |
| Nicotinic acid supplement (g/kg dry matter) | | | - | - | 3.52 | 1.76 |
| | | | | | | |
| *Chemical composition*[b] | | | | | | |
| Dry matter (g/kg) | 368 | 289 | | | | |
| Crude ash (g/kg dry matter) | 37 | 138 | 63 | 51 | 62 | 49 |
| Crude protein (g/kg dry matter) | 80 | 159 | 217 | 219 | 219 | 224 |
| Ether extract (g/kg dry matter) | 32 | 40 | 30 | 30 | 30 | 30 |
| Neutral detergent fiber (g/kg dry matter) | 424 | 514 | 128 | 185 | 130 | 156 |
| Acid detergent fiber (g/kg dry matter) | 223 | 311 | 47 | 47 | 47 | 47 |
| Net energy lactation (MJ/kg dry matter) | 6.4[c] | 6.5[c] | 8.2[d] | 8.3[d] | 8.2[d] | 8.3[d] |
